# Effect of phosphoric acid content on the microstructure and compressive strength of phosphoric acid-based metakaolin geopolymers

**DOI:** 10.1016/j.heliyon.2020.e03853

**Published:** 2020-04-28

**Authors:** Li Gao, Youxiong Zheng, Yan Tang, Jianwei Yu, Xingchang Yu, Bingxin Liu

**Affiliations:** Qinghai Provincial Key Laboratory of New Light Alloys, Qinghai Provincial Engineering Research Center of High Performance Light Metal Alloys and Forming, Qinghai University, Xining, 810016, PR China

**Keywords:** Materials science, Materials chemistry, Metakaolin, Phosphoric acid, Geopolymers, Amorphous phase

## Abstract

The phosphoric acid-based metakaolin geopolymers were prepared by regulating H_3_PO_4_/Al_2_O_3_ ratios. X-ray diffraction (XRD), thermogravimetry and differential scanning calorimeter (TG-DSC), Fourier transform infrared spectroscopy (FTIR) and scanning electron microscopy (SEM) were used to determine the reaction process and phase formation. The results showed that the metakaolin calcined from Kaolinite mainly consisted of quartz crystalline phase and amorphous phase. The diffraction peak for quartz obviously became lower with the increasing of H_3_PO_4_/Al_2_O_3_ ratios. The excessive quartz from metakaolin did not totally take part in the chemical reaction. The polymeric structure of -P-O-Si-O-Al-O constitutes the main building block of phosphoric acid-based metakaolin geopolymeric structure. The optimized compressive strength was 29 ± 2 MPa with H_3_PO_4_/Al_2_O_3_ molar ratio = 1.3:1. The simulation of the total deformation under 29 MPa load and the total heat flux at 1400 °C of the phosphoric acid-based metakaolin geopolymers with H_3_PO_4_/Al_2_O_3_ molar ratio of 1.3:1 based on finite element method verified the failure mechanism and the excellent thermal stability at high temperature.

## 1 Introduction

Geopolymer first recongnized in 1908 is a developing research field for utilizing mineral clays and industrial wastes (Hamdi et al., 2019; Palomo et al., 2014; Provis, 2014). It was utilized to manufacture precast structures, concrete and fixation of toxic metal waste (Ji and Pei, 2019; Davidovits, 1994). The raw materials for producing geopolymers include kaolinite and blast furnace slag (Ozçelik and White, 2016). Through hydrolysis and dissolution, Si and Al are released and can be polymerized to form geopolymers. The main advantages of kaolinite was its good dispersibility and availability of the bond sequence Si–O–Al (Davidovits, 2011).

The geopolymer is prone to generating viscous mortar during processing, so workability in geopolymers is poor and difficult to adjust. This means that the traditional construction industry practices cannot be used for geopolymeric cements. Lloyd et al. (2010) showed that the alkaline geopolymer surfaces are easy to alkali, therefore geopolymer structures will be not able to resist corrosion.

Lloyd (2009) found that for instance geopolymers have a strength loss during use. Furthermore, the thermal stability of the alkali-activated geopolymers was poor due to the phase transformation (Barbosa and MacKenzie, 2003; Provis et al., 2009). In comparison to the alkali-based geopolymers, the acid-based geopolymers exhibit excellent thermal stability (Le-ping et al., 2010; Liu et al., 2012). Currently, research on phosphoric acid-based polymers has attracted growing interest (Morsy et al., 2019). For examples, Sellami et al. (2019) prepared phosphoric acid-based metakaolin geopolymer with conductivity of 10^−7^ S/cm at temperatures above 625 °C, which can be used as a good thermal insulator. Mathivet et al. (2019) performed a thorough study on structural evolutions of acid-based geopolymers. However, there have been few studies about compressive strength of acid-based geopolymers.

In this work, we have successfully synthesized phosphoric acid-based geopolymers by kaolinite and phosphoric acid. The effect of H_3_PO_4_/Al_2_O_3_ molar ratio on compressive strength of the synthesized geopolymers was investigated. The reaction mechanisms and characteristics of the acid-based geopolymers were studied by means of XRD, FTIR, TG-DSC and SEM.

## 2 Experimental

### 2.1 Materials

Kaolin (Al_2_O_3_·2SiO_2_·2H_2_O) was purchased from kaolin company, China. Distilled water and phosphoric acid (85 wt%, AR) was used in the experiment.

### 2.2 Sample preparation

Metakaolin was prepared by calcining Kaolin at 800 °C for 120 min to dehydrate. The phosphoric acid solution was mixed by stirring in a mixer for 30 min to obtain a homogeneous slurry with molar ratios of H_3_PO_4_/Al_2_O_3_ from 1.0:1 to 1.4:1. The mass ratio of solid to liquid was 1:1 in the slurry. The slurry was poured into cubic molds (60 mm × 60 mm× 60 mm), and the molds were sealed to suppress water loss. All specimens were cured at ambient temperature for 7 days. After demolded, the samples were cut into cubic shape with dimension of 20 mm × 20 mm ×20 mm.

### 2.3 Characterization

X-ray fluorescence (XRF, EDX 1800B) was used to analyze chemical compositions of samples. The average particle size of metakaolin was tested by laser particle size analyzer (Mastersize 2000). Compressive strength was measured on cubic specimens with dimension of 20 mm × 20 mm × 20 mm and loaded with a crosshead speed of 0.5 mm/min by a mechanical testing machine (SHIMADZU AG-2000G). X-ray diffraction (D8 advance A25, Cu Kα) was employed to monitor the phase formation in the 2θ range from 10 to 80° with a step size of 0.02°. Differential scanning calorimetry (DSC Q200) and thermogravimetry (TG STA449F3) were used to investigate the thermal stability. TG-DSC experiment was carried out with a heating rate of 10 °C/min. FT-IR (VERTEX 70V) was used to analyze chemical bonding state in the structure. Scanning electron microscopy (SEM, JSM 6700F, JEOL) was used to analyze the microstructures of geopolymers after sputtering gold coating on the surfaces.

## 3 Results and discussion

The average particle size of metakaolin by calcining Kaolin was 15.1 μm. The chemical compositions of kaolin and metakaolin determined by XRF were shown in Table 1.

**Table 1:** Chemical compositions of kaolin and metakaolin.

Raw materials	Chemical composition (wt/%)								
	SiO_2_	Al_2_O_3_	SO_3_	K_2_O	Fe_2_O_3_	TiO_2_	ZnO	P_2_O_5_	MgO
Kaolin	56.63	34.74	4.61	1.46	0.82	0.59	0.32	0.27	0.17
Metakaolin	57.06	37.80	0.61	1.72	0.94	0.60	0.34	0.27	0.12

### 3.1 XRD analysis

**Figure 1:**
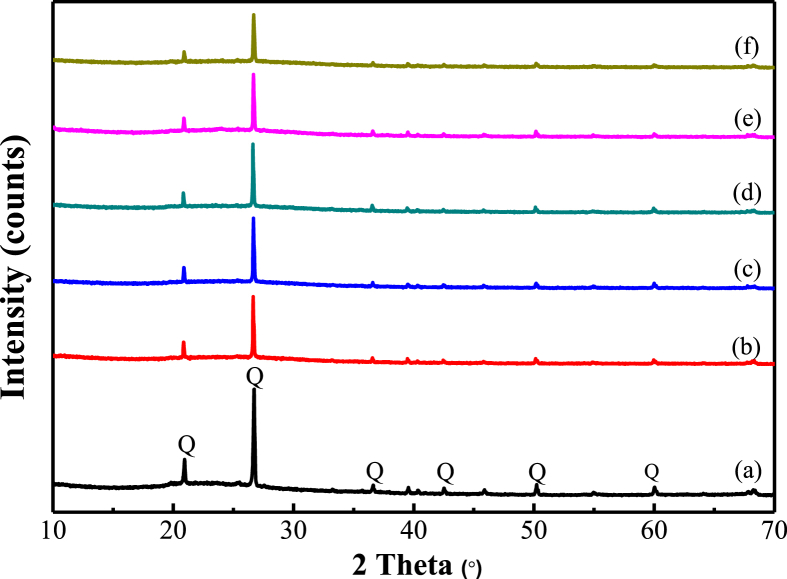
XRD patterns of metakaolin and geopolymers with different H_3_PO_4_/Al_2_O_3_ ratios. Q, Quartz (PDF # 79-1906). (a) Metakaolin; (b) H_3_PO_4_/Al_2_O_3_ = 1.0:1; (c) H_3_PO_4_/Al_2_O_3_ = 1.1:1; (d)H_3_PO_4_/Al_2_O_3_ = 1.2:1; (e) H_3_PO_4_/Al_2_O_3_ = 1.3:1; (f) H_3_PO_4_/Al_2_O_3_ = 1.4:1.

Figure 1 shows the XRD patterns of metakaolin and geopolymers with different H_3_PO_4_/Al_2_O_3_ ratios. Pattern (a) of metakaolin reveals that there were quartz crystalline phase and amorphous phase in metakaolin. As for the XRD patterns of geopolymers with different H_3_PO_4_/Al_2_O_3_ ratios (Pattern (b) ~ Pattern (f)), even though there still were quartz crystalline phase and amorphous phase in each sample, the diffraction peak for quartz obviously became lower with the increasing of H_3_PO_4_/Al_2_O_3_ ratios. The remaining quartz peak indicated that excessive quartz from metakaolin did not take part in the chemical reaction. Other impurities were not found.

### 3.2 Compressive strength

**Figure 2:**
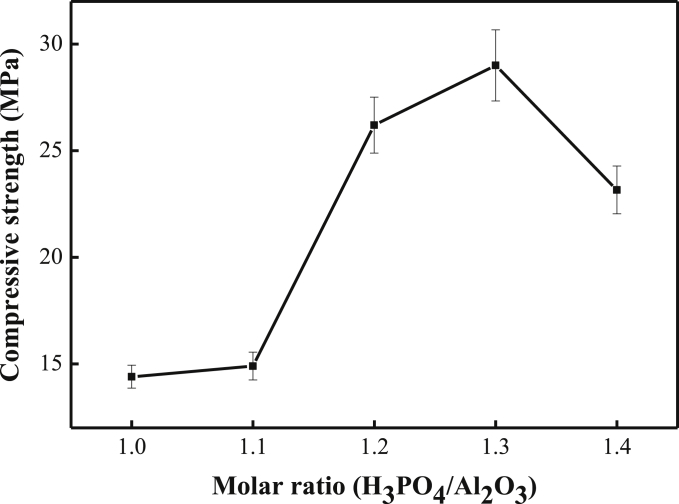
Compressive strength of the geopolymers with different H_3_PO_4_/Al_2_O_3_ molar ratios.

Figure 2 illustrates the compressive strength of the geopolymers with different H_3_PO_4_/Al_2_O_3_ molar ratios. The compressive strength of phosphoric acid-based geopolymers were 14.4 MPa, 14.9 MPa, 26.2 MPa, 29 MPa and 23.16 MPa, respectively, when the molar ratio of H_3_PO_4_/Al_2_O_3_ was 1:1 to 1.4:1. As shown in Figure 2, the optimized compressive strength was 29 ± 2 MPa with H_3_PO_4_/Al_2_O_3_ molar ratio = 1.3. The compressive strength with 1.2–1.4 H_3_PO_4_/Al_2_O_3_ molar ratio of geopolymers was about twice of those of the geopolymers with 1.0–1.1 H_3_PO_4_/Al_2_O_3_ molar ratio. The amount of elicitor acid was not enough to excite the metakaolin when the H_3_PO_4_/Al_2_O_3_ molar ratio was less than 1.2, while excessive phosphoric acid made the samples difficult to cure when the H_3_PO_4_/Al_2_O_3_ molar ratio was larger than 1.4.

**Figure 3:**
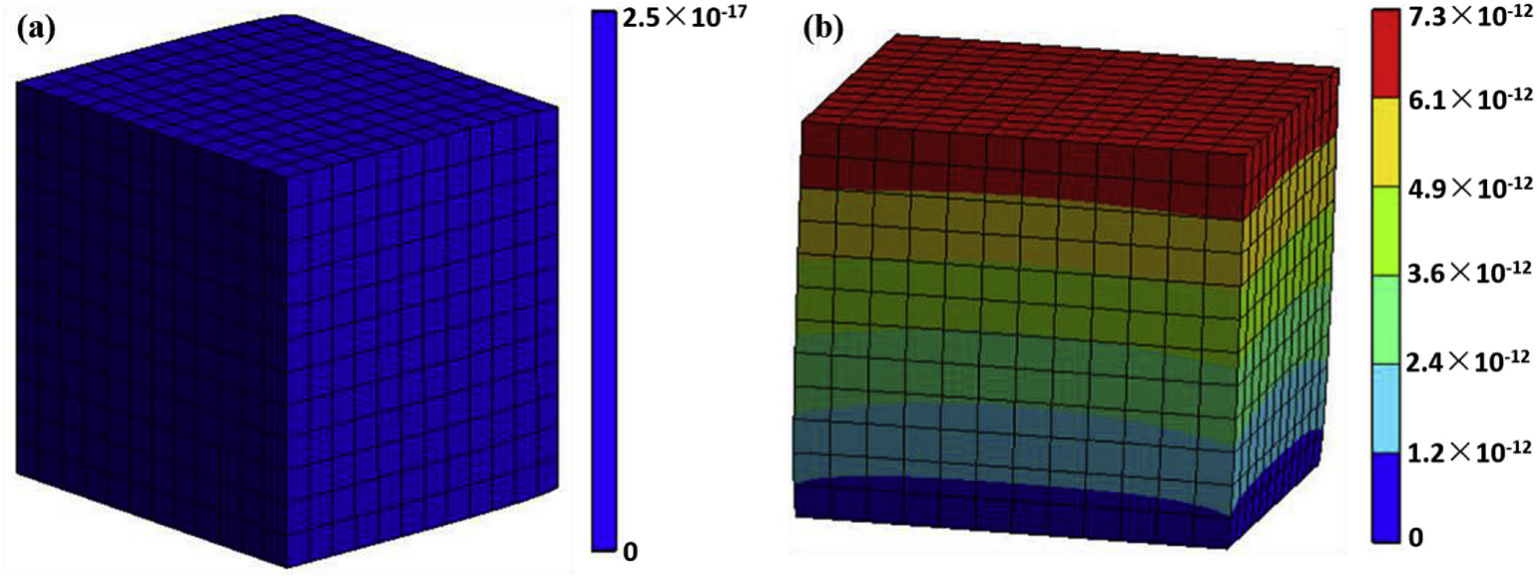
Finite element analysis of total deformation before and after compression of geopolymers with H_3_PO_4_/Al_2_O_3_ molar ratio of 1.3. (a) 0 MPa; (b) 29 MPa.

Figure 3 is the finite element analysis about total deformation of the geopolymers with different loads. The simulation process was performed on the ANSYS Workbench simulation platform. As shown in Figure 3 (a) and (b), the total deformation of the geopolymer gradually was increased with the increasing of the load. The deformation of the surface (the surface under direct stress) was the largest compared with other parts (Figure 3 (b)). This indicated that when the load reached the limit that the polymer can bear, the surface of the sample first cracked and gradually spreaded to other parts of the sample, eventually leading to the destruction of the whole sample.

### 3.3 SEM analysis

**Figure 4:**
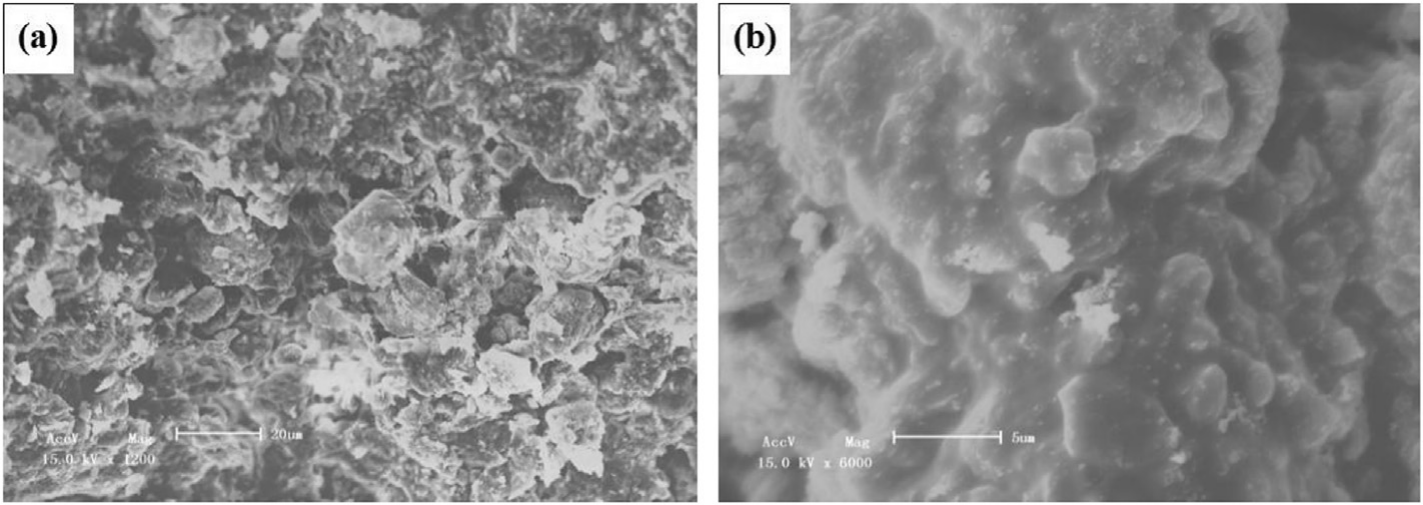
SEM micrographs of geopolymer with H_3_PO_4_/Al_2_O_3_ of 1.3 at different magnifications. (a)×1200, (b) ×6000.

Figure 4 indicates the microstructure of the phosphoric acid-based geopolymers with H_3_PO_4_/Al_2_O_3_ molar ratio of 1.3. The geopolymer was mainly composed of lamellar, as observed in Figure 4 (a), and the dense amorphous glassy geopolymer matrices and some particle can be clearly seen in Figure 4 (b). The particles were probably the quartz crystals existed in the samples, because quartz from metakaolin did not dissolve totally into the gel phase according to Figure 1(XRD).

### 3.4 FT-IR analysis

**Figure 5:**
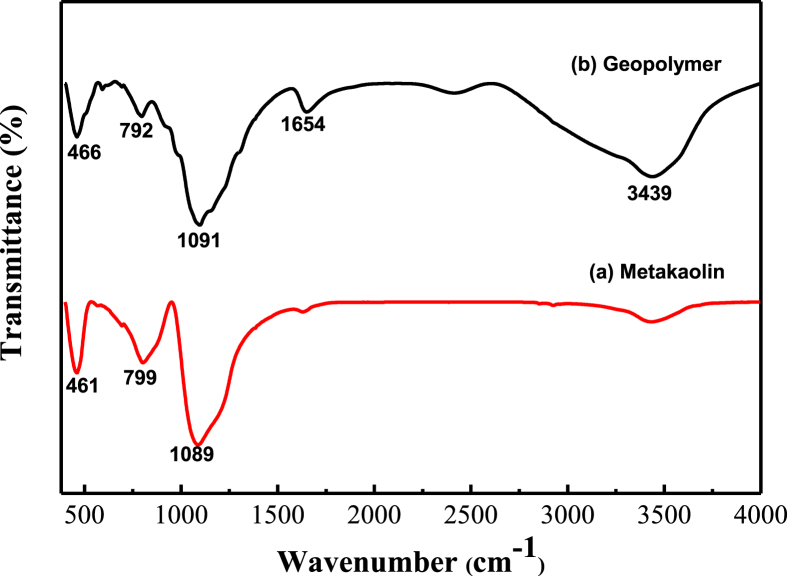
FT-IR spectra of (a) metakaolin and (b) geopolymer.

Figure 5 illustrates FT-IR spectra of metakaolin and phosphoric acid-based geopolymer with H_3_PO_4_/Al_2_O_3_ molar ratio = 1.3. The presence of three key vibrational modes in FT-IR spectra of metakaolin, 1089 cm^−1^ of Si–O–Si asymmetric stretching, 799 cm^−1^ of Al–O–Si and 461 cm^−1^ of Si–O bending, indicated the formation of metakaolin after calcining kaolinite at 800 °C (Wang et al., 2005; Douiri et al., 2014). The band of P–O stretching vibration was normally found at 1087–1335 cm^−1^ in IR spectra of the geopolymer, so the broad absorption band around 933–1335 cm^−1^ corresponded to the stretching vibration of P–O–Si–O. The band around 1600–1740 cm^−1^ arised from the adsorbed water and P–O group of stretching and bending vibration. The peaks 792 cm^−1^ and 466 cm^−1^ were due to Al–O–Si vibration. The broad absorption band around 3400-3700 cm^−1^ was corresponded to the vibration of hydroxyl (OH) groups and H_2_O molecules (Smith, 1999). Based on above experimental results and previous studies (Liu et al., 2012; Cao et al., 2005), the geopolymerization mechanism in the reaction system of phosphoric acid and metakaolin was believed to be a “bonding reaction” among the P–O tetrahedral units of phosphoric acid solution, the Si–O tetrahedral units and the Al–O layer of metakaolin. Therefore, the -P-O-Si-O-Al-O structure formed during geopolymerization constitutes the main structural unit of phosphoric acid-based metakaolin geopolymer structure.

### 3.5 Thermal analysis

**Figure 6:**
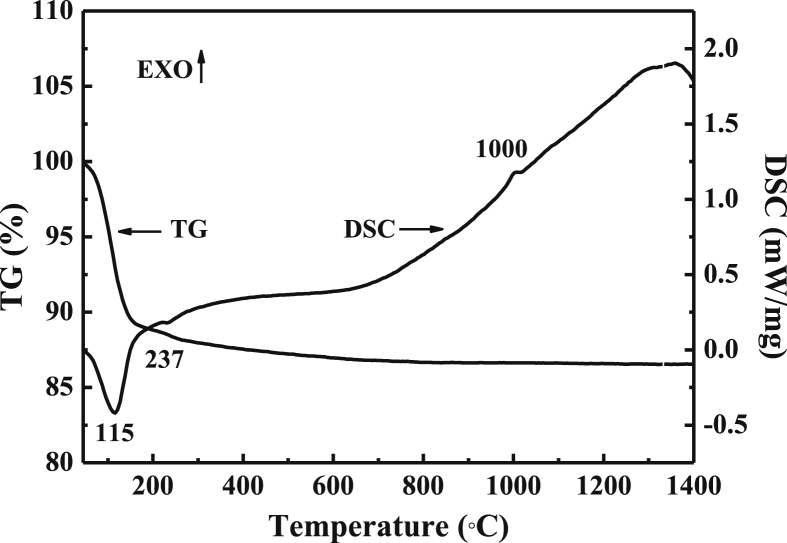
TG-DSC curves of the geopolymer with H_3_PO_4_/Al_2_O_3_ molar ratio of 1.3.

TG-DSC curves in the temperature range from 50 to 1450 °C of the geopolymers are shown in Figure 6. A clear weight loss of about 10.5% in the temperature range 50–200 °C was observed and the corresponding endothermic peak was found in the DSC curve at about 115 °C, which was ascribed to the removal of absorbed water and hydroxyl groups. Another small weight loss of 0.5% was observed between 200 and 250 °C due to dehydroxylation. The exothermic peak between 995 and 1050 °C and the exothermic peak between 1300 and 1400 °C were attributed to formation of mullite and transformation of cristobalite from quartz. Despite the one exothermic peak, the mass keeped almost unchanged between 600 and 1450 °C. Therefore, the phosphoric acid-based metakaolin geopolymers exhibited an excellent thermal stability and were stable at temperature up to 1400 °C.

**Figure 7:**
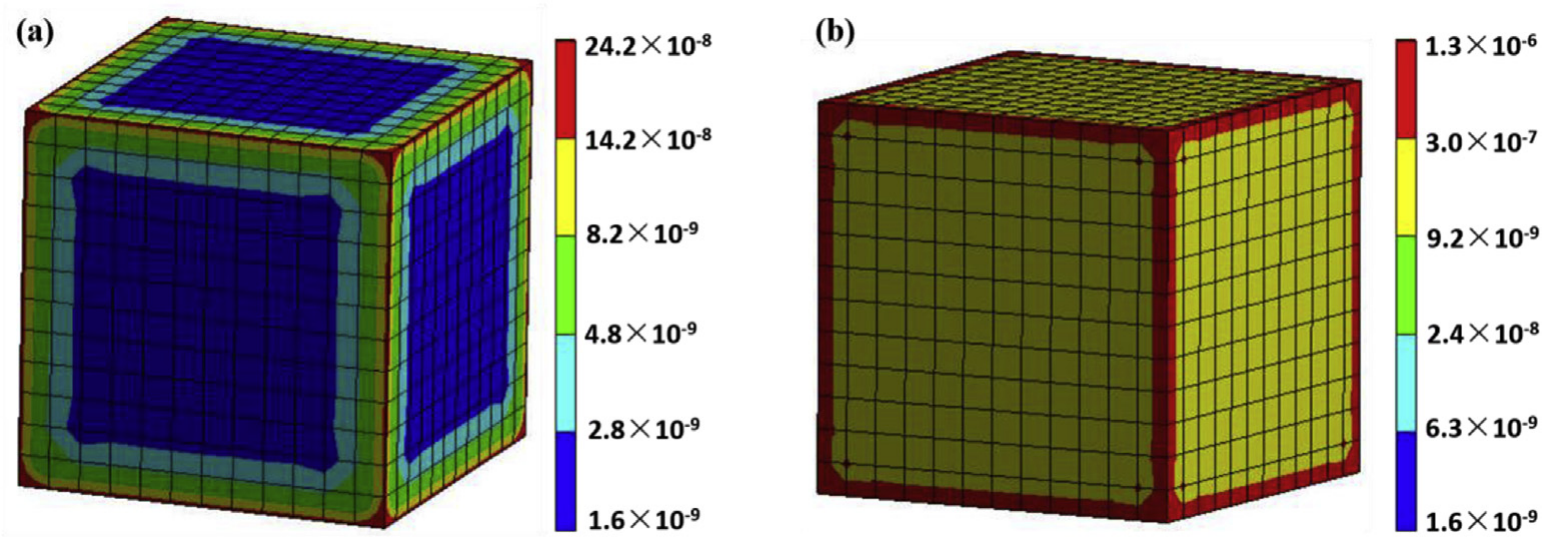
Finite element analysis of the total heat flux at different temperature of geopolymers with H_3_PO_4_/Al_2_O_3_ molar ratio of 1.3. (a) 25 °C; (b) 1400 °C.

Figure 7 is the finite element analysis about the total heat flux at different temperature of geopolymers with H_3_PO_4_/Al_2_O_3_ molar ratio of 1.3. The heat flux at the edge portion of the geopolymers was larger relative to other parts, which may be due to uneven stress inside the cube. The overall heat flux of the geopolymers was much larger than the heat flux at room temperature (Figure 7 (a)) when the temperature reached 1400 °C (Figure 7 (b)), but its highest heat flux was still low. It was consistented with the analysis results of TG-DSC, indicating that the phosphoric acid-based metakaolin geopolymers were provided with superior thermal stability at high temperature.

## 4 Conclusion

Phosphoric acid-based metakaolin geopolymers were synthesized by metakaolin, phosphoric acid and distilled water, and the -P-O-Si-O-Al-O structure formed after geopolymer formation. The acid-based metakaolin geopolymers were composed of glassy amorphous phase and small amounts of quartz. The phase composition of these geopolymers consisted of amorphous phase and minor amounts of quartz. Excessive quartz from metakaolin did not totally take part in the chemical reaction. The intensities of quartz peak decreased when H_3_PO_4_/Al_2_O_3_ ratios increased, therefore the excitation effect of acid activator is observed. The optimized compressive strength was 29 ± 2 MPa with H_3_PO_4_/Al_2_O_3_ molar ratio = 1.3. The results of the finite element analysis of total deformation indicated that the geopolymer first cracked from the surface under load and gradually spread to other parts until the sample was completely destroyed. The excellent thermal stability at 1400 °C of the geopolymers was proved by the finite element analysis of total heat flux of the geopolymers.

## Declarations

### Author contribution statement

Li Gao: Conceived and designed the experiments; Analyzed and interpreted the data; Contributed reagents, materials, analysis tools or data; Wrote the paper.

Youxiong Zheng: Performed the experiments; Contributed reagents, materials, analysis tools or data.

Yan Tang: Analyzed and interpreted the data.

Jianwei Yu, Xingchang Yu: Performed the experiments.

Bingxin Liu: Conceived and designed the experiments.

### Funding statement

This work was supported by the 10.13039/501100012579Natural Science Foundation of Qinghai Province, China (2020-ZJ-764); 10.13039/501100001809National Natural Science Foundation of China, China (21804078); Thousand Talents Program of Qinghai Province.

### Competing interest statement

The authors declare no conflict of interest.

### Additional information

No additional information is available for this paper.
